# Supplementary material for: Mapping recurrent mosaic copy number variation in human neurons
Source: Nat Commun. 2024 May 17;15:4220. doi: 10.1038/s41467-024-48392-0 (PMC11101435; doi:10.1038/s41467-024-48392-0)
Supplement: Supplementary file 1 — Supplementary Information [file 41467_2024_48392_MOESM1_ESM.pdf]

Supplementary Figures

Mapping the Complex Genetic Landscape of Human Neurons

Chen Sun[1,#] , Kunal Kathuria[2,#], Sarah B Emery[3], ByungJun Kim[1], Ian E. Burbulis[4,5], Joo Heon Shin[2], Brain Somatic Mosaicism Network, Daniel R. Weinberger[2,6,7], John V. Moran[3,8], Jeffrey M. Kidd[1,3], Ryan E. Mills[1,3*], Michael J. McConnell[2*]
[1]Department of Computational Medicine and Bioinformatics, University of Michigan Medical School, 100 Washtenaw Avenue, Ann Arbor, MI 48109, USA.
[2]Lieber Institute for Brain Development, 855 North Wolfe Street, Baltimore, MD 21205, USA.
[3]Department of Human Genetics, University of Michigan Medical School, 1241 East Catherine Street, Ann Arbor, MI 48109, USA.
[4]Department of Biochemistry and Molecular Genetics, University of Virginia, School of Medicine, Charlottesville, VA 22902, USA
[5]Facultad de Medicina y Ciencia, Universidad San Sebastián, Sede de la Patagonia, Puerto Montt, Chile.
[6]Department of Psychiatry and Behavioral Sciences and Neuroscience, Johns Hopkins School of Medicine, 600 North Wolfe Street, Baltimore, MD 21287, USA.
[7]McKusick-Nathans Institute of Genetic Medicine, Johns Hopkins School of Medicine, 733 North Broadway, Baltimore, MD 21230, USA.
[8]Department of Internal Medicine, University of Michigan Medical School, 1500 East Medical Center Drive, Ann Arbor, MI 48109, USA.

# These authors contributed equally
* Communication to mikemc@libd.org, remills@umich.edu

Supplementary Fig. 1 1

Supplementary Fig. 2 2

Supplementary Fig. 3 3

Supplementary Fig. 4 4

Supplementary Fig. 5 5

Supplementary Fig. 6 6

Supplementary Fig. 7 7

Supplementary Fig. 8 8

Supplementary Figure 9 9

Supplementary Figure 10 10

Supplementary Figure 11 11

Supplementary Figure 12 12

Supplementary Figure 13 13

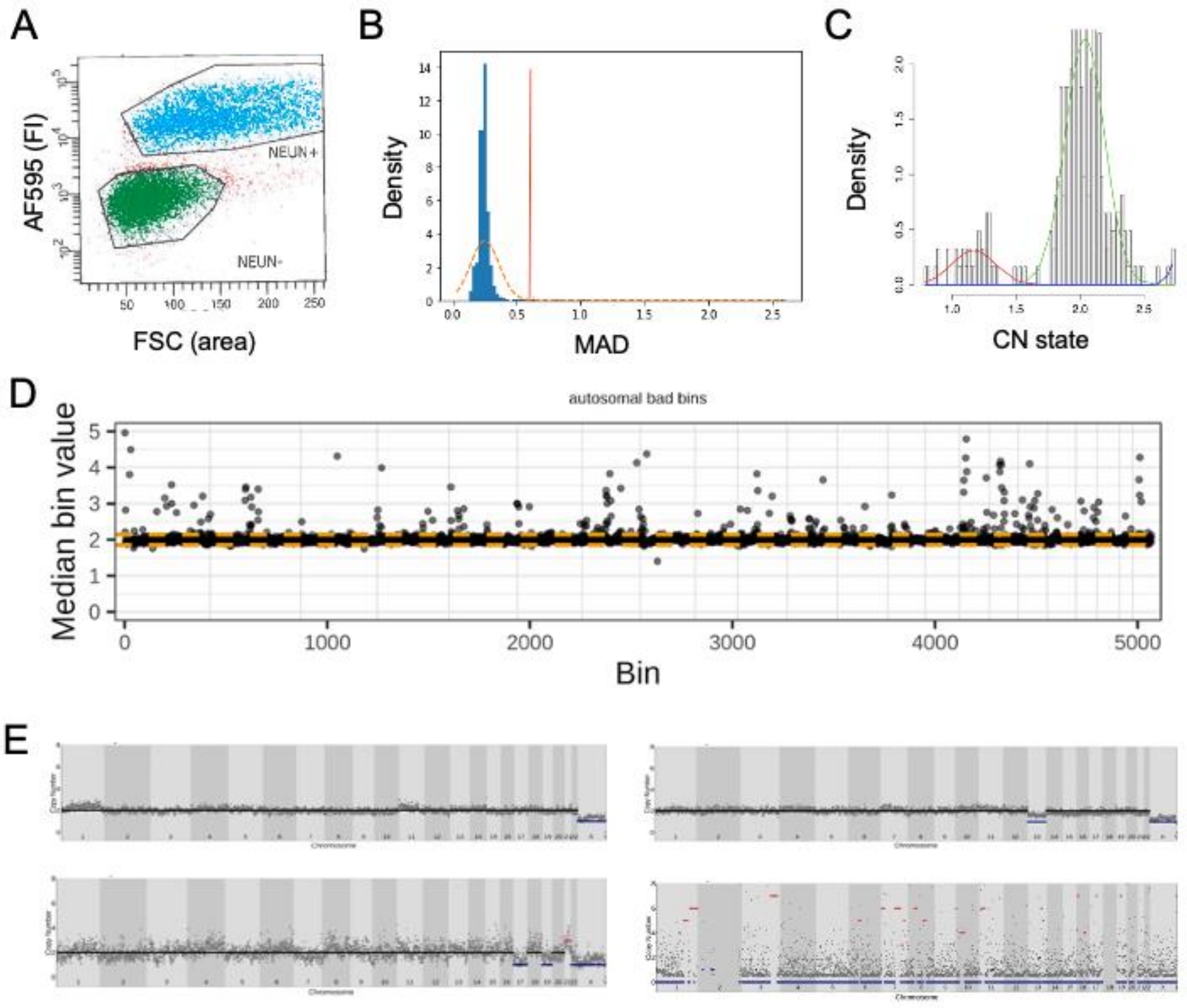

Supplementary Figure 1

Optimization of Ginkgo for read-depth-based CNV calls (**A**) NeuN+ (AF595) nuclei were isolated by FANS. **(B)** Mean absolute deviation (MAD) score distribution (based on bin copy numbers) excluded 19 of 2,125 neurons (with MAD > 3 standard deviations away from mean). (**C**) Thresholds for calling putative CNVs were set using a GMM based on 585 cells obtained from the 5 control individuals studied in Chronister, et al. (1.63 for deletions, 2.43 for duplications). (**D**) Tukey's rule was applied to median copy numbers for all genomic bins across all neurons in our dataset to yield 308 additional outlier bins in addition to Ginkgo's original 29 that were excluded from further analysis. (**E**) Four cells passed the MAD cutoff but were curated manually due to ambiguous copy-number patterns, including 1 that did not pass the read-count filter due to concentration of reads on Chr2 (bottom right).

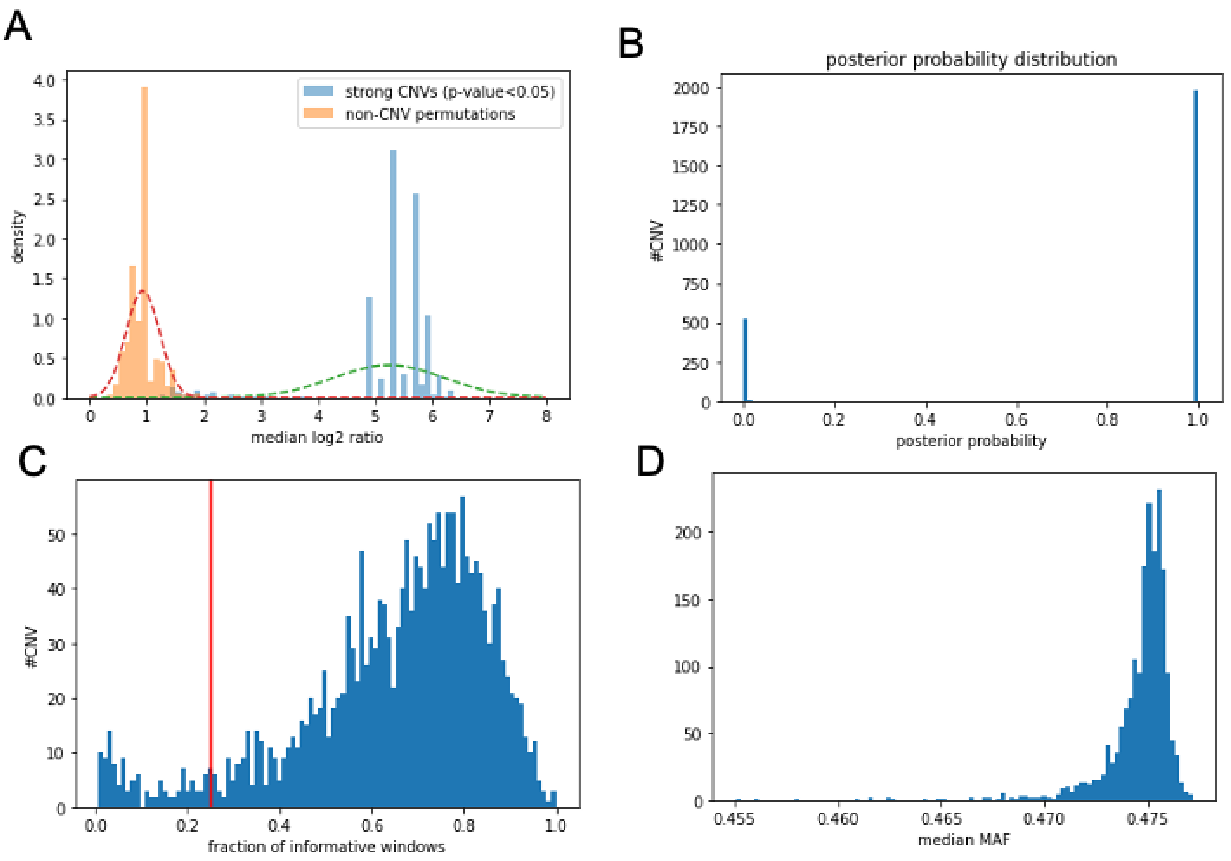

Supplementary Figure 2

Naïve Bayesian-based pipeline to filter CNVs. (**A**) We labeled CNV calls as “strong” based on an empirical p-value, which is derived from the median absolute log2 ratio of the windows within the CNV regions. Then we derived a Gaussian mixture model of strong calls and 100 non-CNV set permutations. (**B**) Using the median absolute log2 ratios of the two datasets as the training data, we estimated the parameters of the Gaussians and predicted the posterior probability that a candidate CNV belonged to a specific CNV distribution. (**C**) We filter out deletion calls where more than 75% of its het-SNP windows contained fewer than 3 informative reads which precludes an accurate haplotype assessment. **(D)** An analysis of the minor allele frequency (MAF) of germline heterozygous SNPs falling within detected somatic CNVs show patterns that are consistent with diploid haplotypes, with all falling within ~5% of the expected 50% allele ratio, indicating these are unlikely to be germline CNVs.

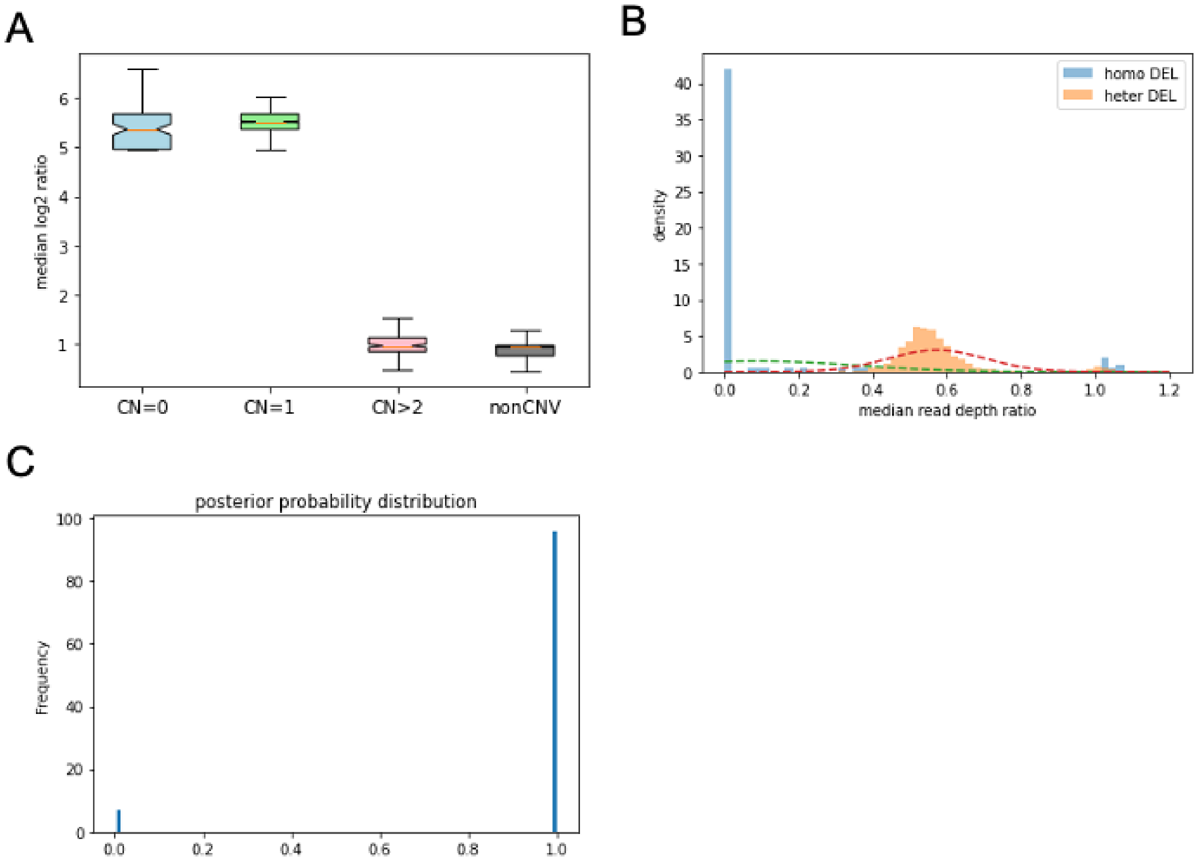

Supplementary Figure 3

Homozygous deletions and duplications are more challenging to validate using SCOVAL. (A) The median absolute log2 ratio of informative reads in candidate homozygous deletions informative reads are similar to heterozygous deletions. The median absolute log2 ratio of duplications are not significantly different from randomly sampled non-CNV regions. (B) Derived Gaussian mixture model from median read depth ratios between homozygous and heterozygous deletions. (C) Posterior probability for putative homozygous deletions using a naive Bayesian classifier on the Gaussian mixture model from the initial heterozygous and homozygous deletion calls.

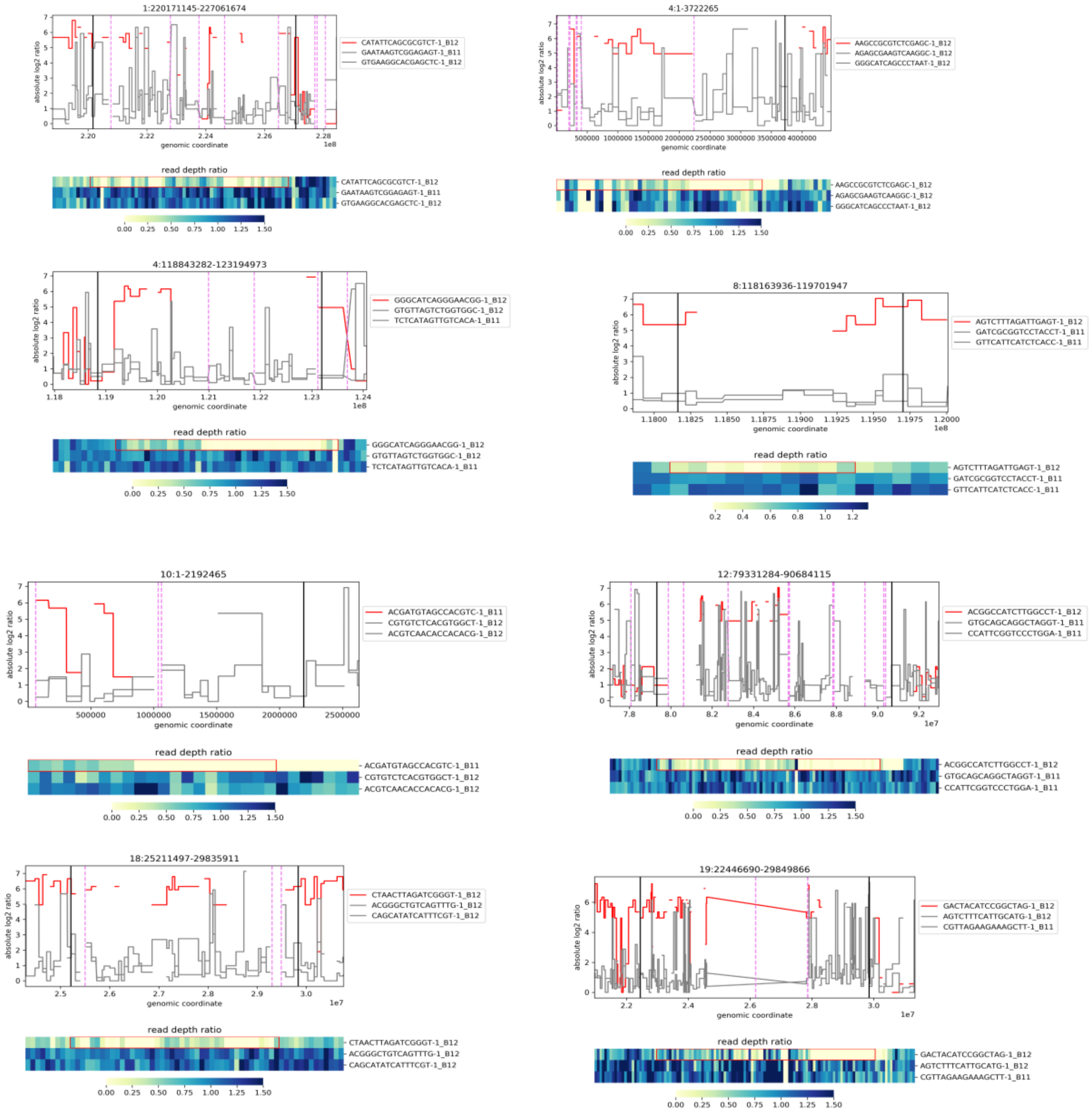

Supplementary Figure 4

Heterozygous deletions miscalled as homozygous deletions. We identified 8 homozygous deletion calls from Ginkgo with read depth and allele ratio characteristics consistent with heterozygous deletions. The upper panel for each figure is the absolute log2 ratio. Red line indicates the cell with CNV and the gray lines represent two random background cells. The bottom panel is the read depth ratio. The first row is for the cell with the candidate CNV, supplemented in rows two and three with randomly chosen cells as background.

A

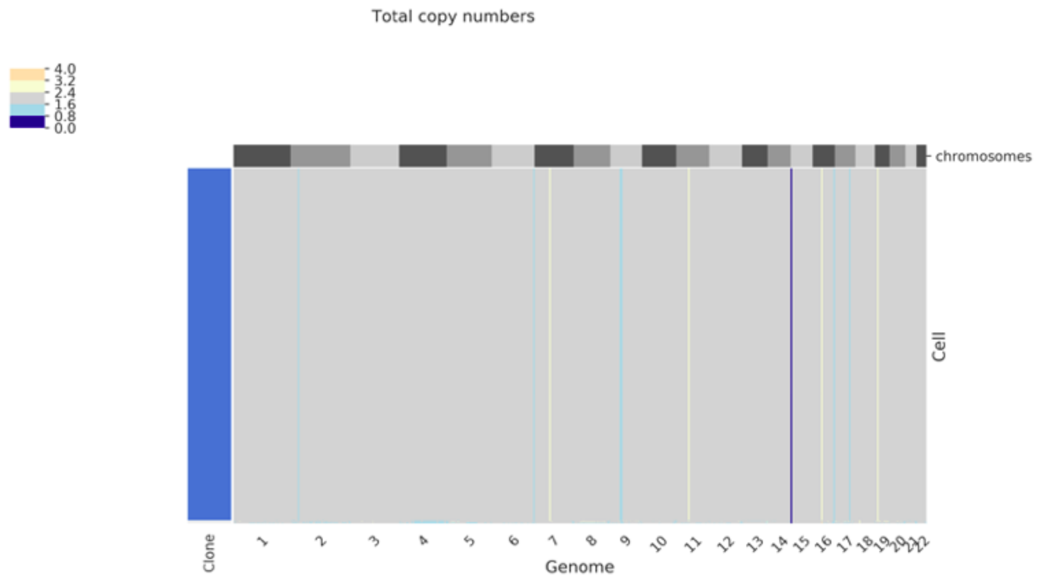

B

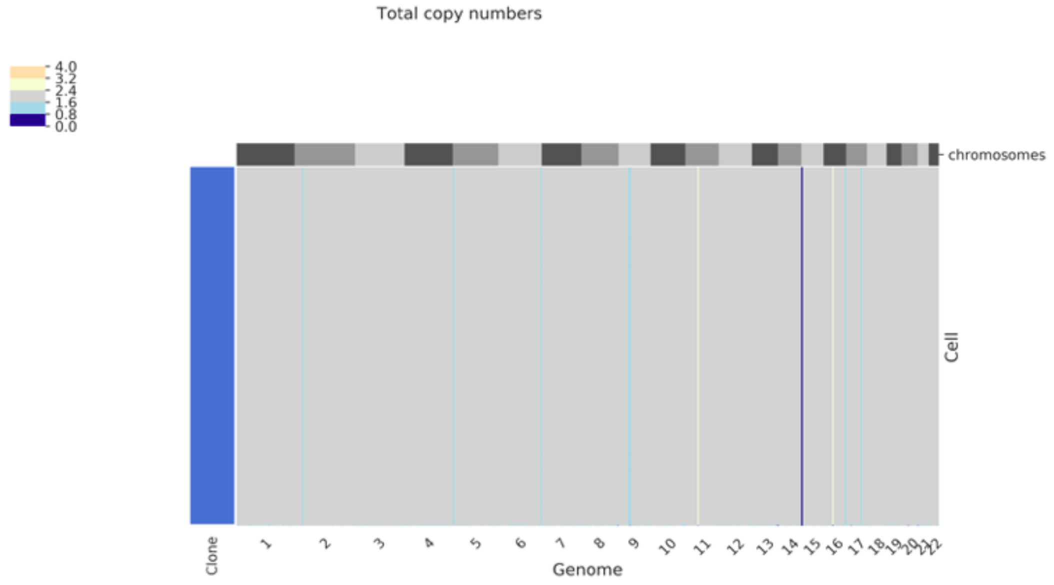

Supplementary Figure 5

Benchmarking CNV detection with CHISEL. Output of CHISEL for our single-cell sequencing data in (**A**) batch B11 and (**B**) batch B12 using diploid=2 parameters. The majority of reported CNVs were reported in all cells and overlap outlier genomic bins that were removed.

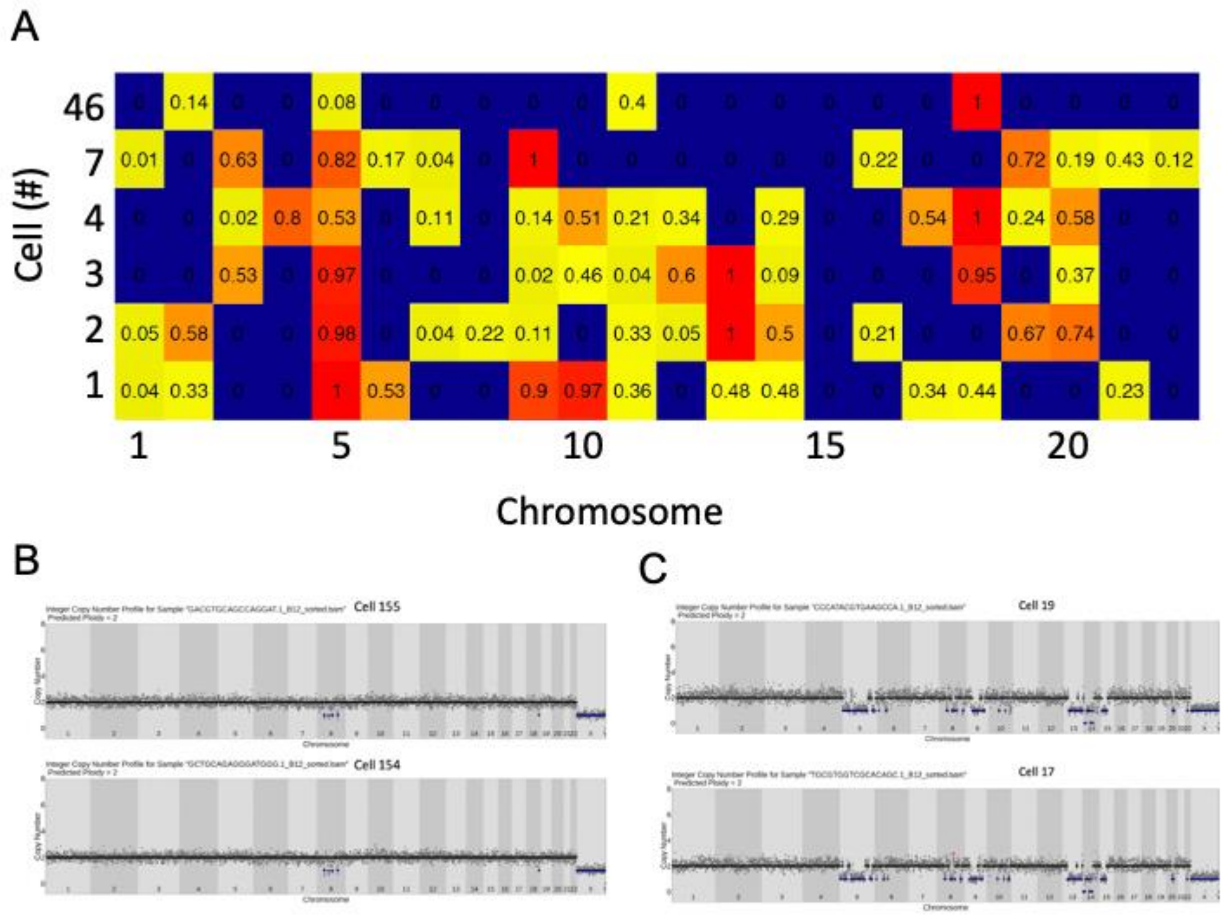

Supplementary Figure 6

Aneuploid neurons and technical replicates. (**A**) Aneuploid neurons from Figure 2D. Each row is an aneuploid neuron, the percent of each chromosome's LOH is indicated by text and color (yellow < 0.5, orange > 0.5, red =1). (**B, C**) Two pairs of libraries produced nearly identical CNV profiles. We interpret these as technical replicates wherein two barcodes were paired with a single nucleus.

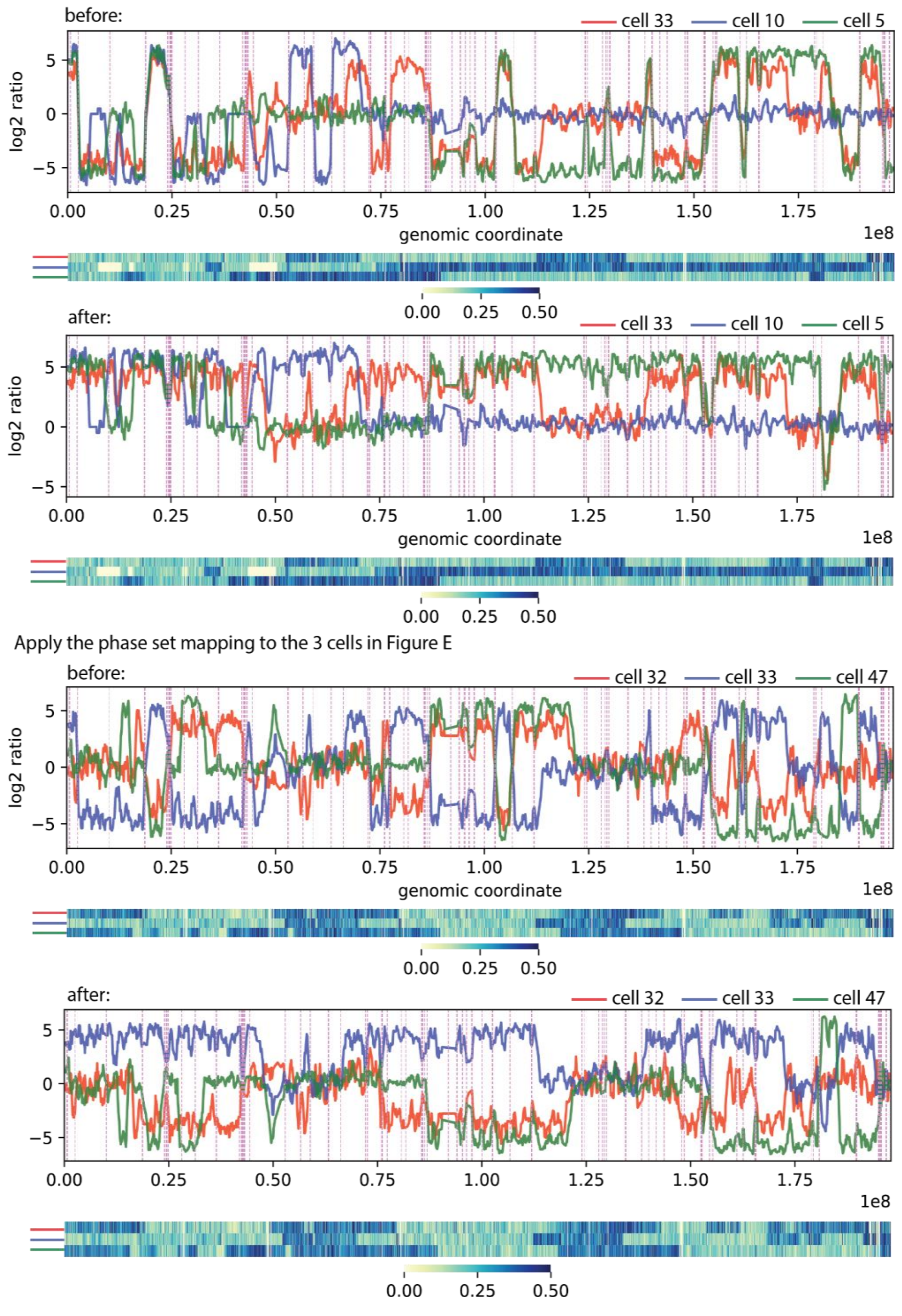

Supplementary Figure 7

Reconstruction of Chromosome 3 haplotypes using overlapping heterozygous deletions in 3 cells. We generated extended phase blocks using three CNV neurons (cells #33, #10, and #5) that contained overlapping deletions that in aggregate cover the full-length of Chromosome 3 in order to determine phasing at chromosome level. These were used to reconstruct the haplotype of 3 cells reported in Figure 2E.

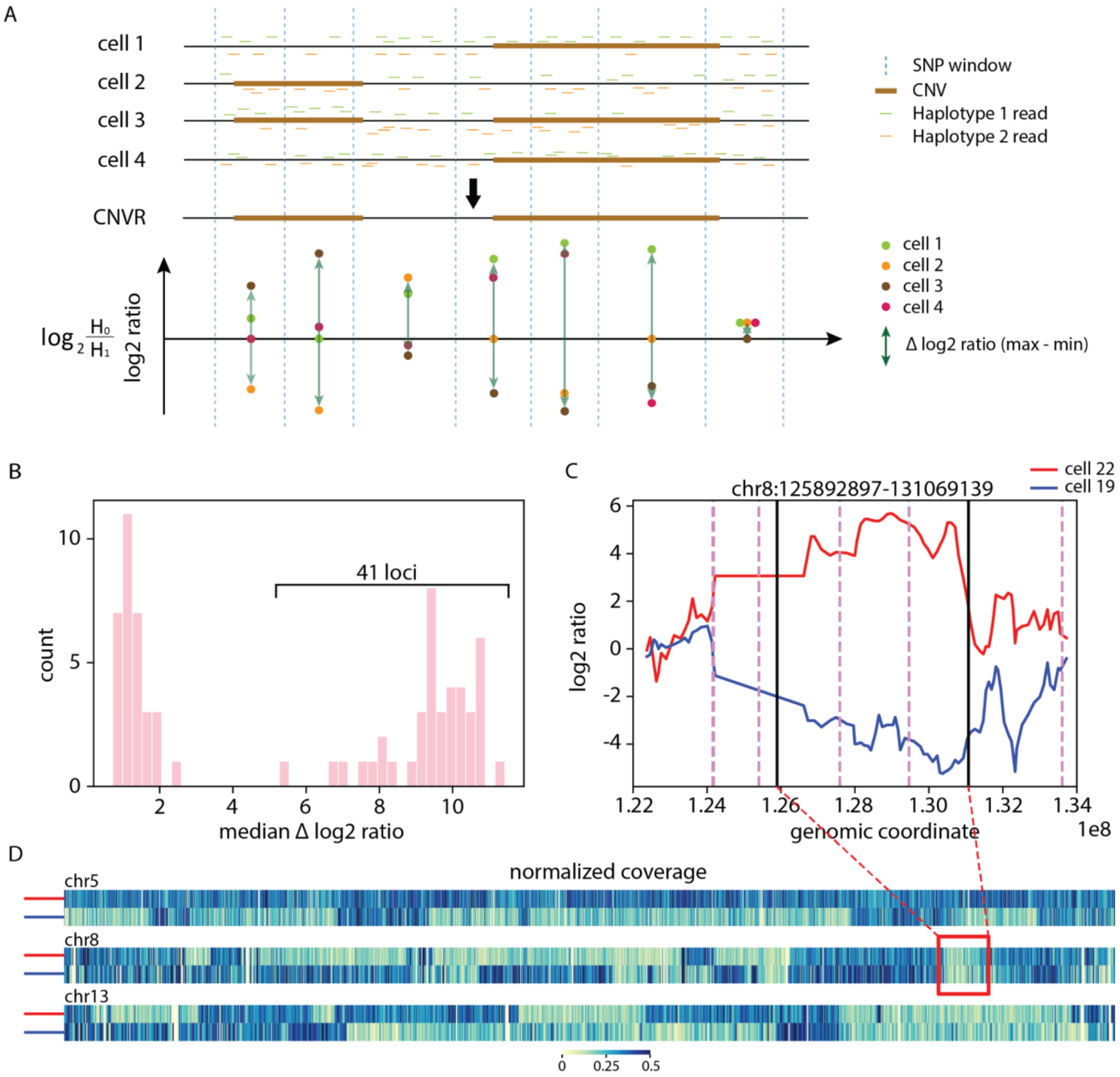

Supplementary Figure 8

CNVs sharing the same location are on different haplotypes. (**A**) We derived a min-max median delta log2 ratio to determine whether CNVRs likely reside on the same haplotype. (**B**) There are two apparent distributions of delta log2 ratio values. CNVs from 41 CNVRs with higher median delta log2 ratio likely occurred on different haplotypes. (**C**) Two cells (#22 and #19) both exhibit CNVs with the same location on Chr8, but show allelic ratios consistent with residing on different haplotypes. (**D**) An examination of CNVs on other chromosomes in these cells further indicate that these shared CNVs are not clonally derived.

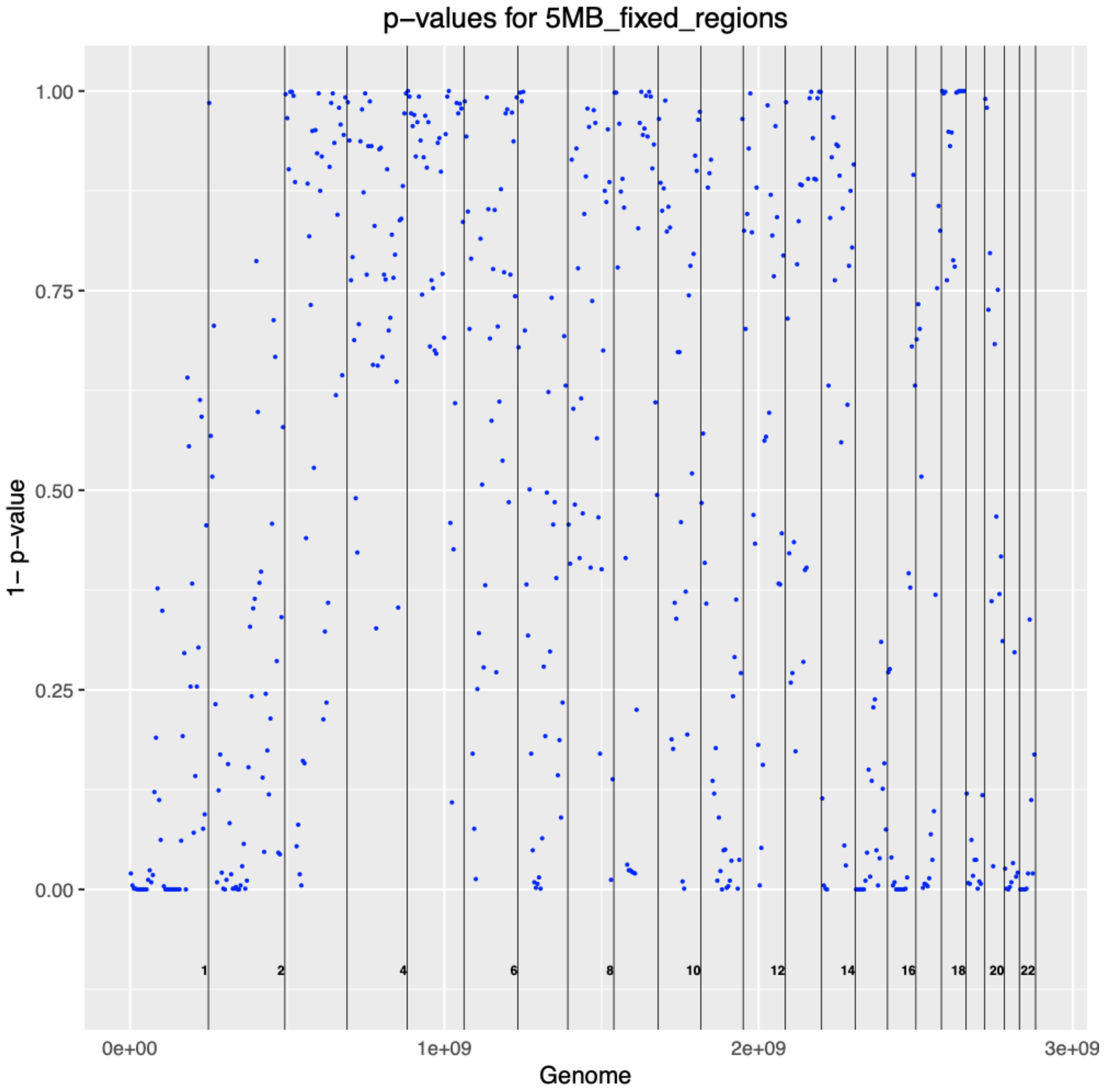

Supplementary Figure 9

Genome-wide identification of hotspots and cold spots. Regional significance (1 minus p-value of number of CNV hits in a 5Mb region compared to random synthetic data) is plotted for all 5Mb genomic regions. Hotspots and cold spots shown in Fig. 3C. were identified using values > .95 and < .01 respectively.

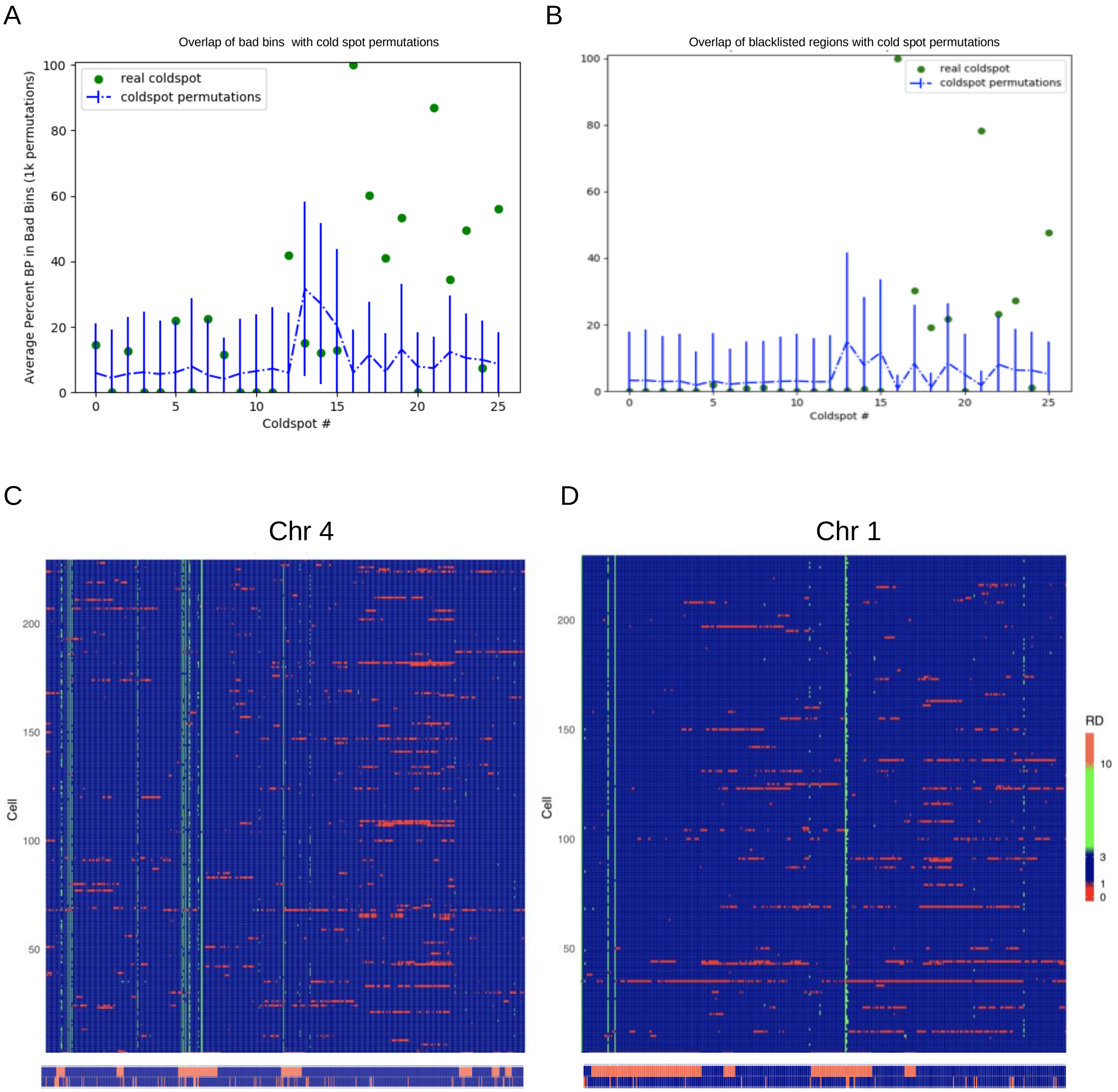

Supplementary Figure 10

Filtering of cold spots based on unmappable genomic regions (**A,B**) Percentage of cold spots occupied by bad bins and by blacklisted regions identified by ENCODE respectively (green) compared to median of same quantity for cold spot permutations in control regions (blue). Cold spots registering high unmappable content (p-value < .05 cutoff) were filtered out (see **Methods**). (**C,D**) Schematic overview showing correlation of read depth (upper panels), cold spots, and bad bins (lower panels) for all CNV neurons across all genomic bins for 2 different chromosomes.

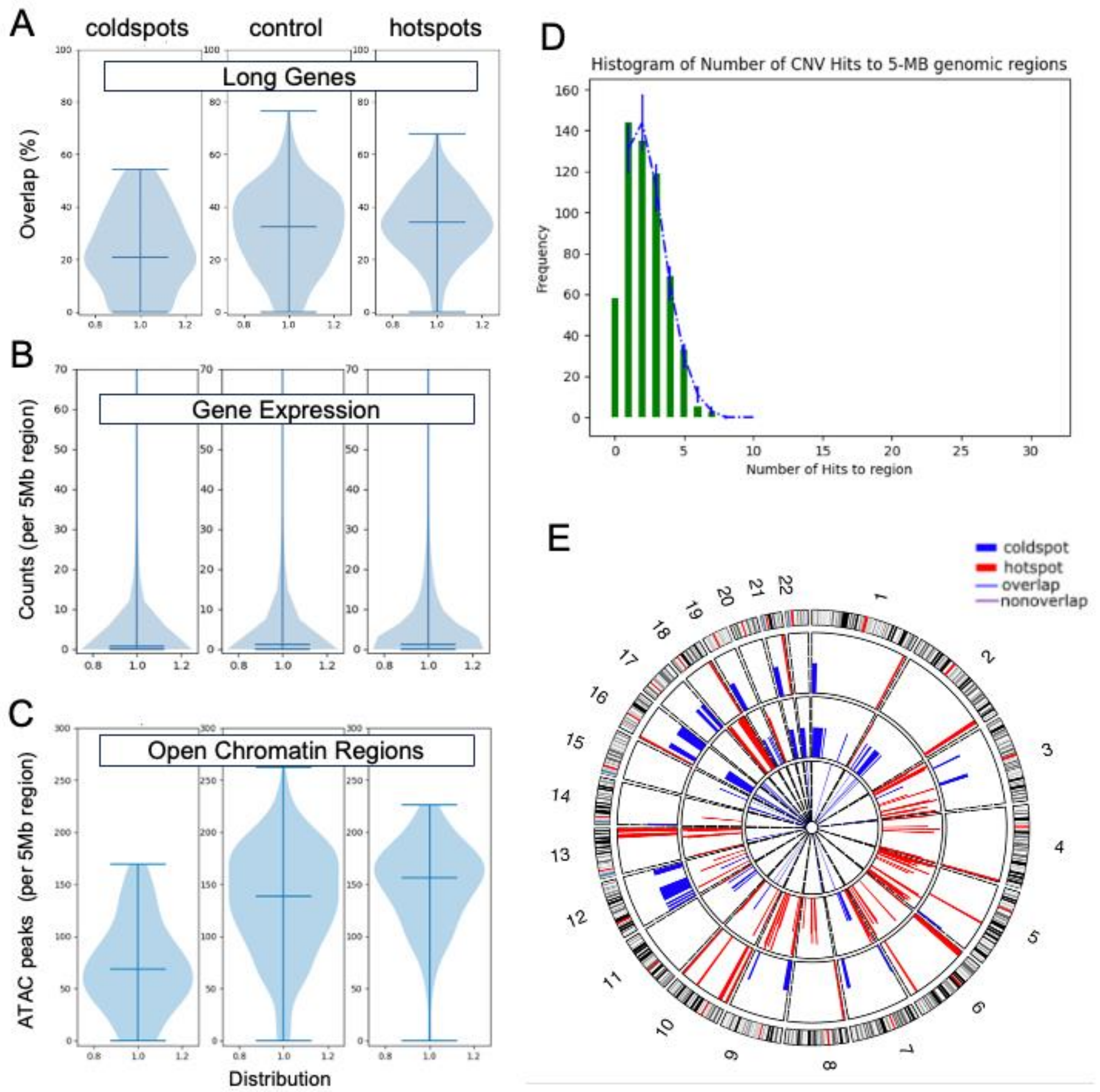

Supplementary Figure 11

Features of hotspots and cold spots. (A, B, C) Features of cold spots (left), control regions (middle), and hotspots (right) are presented in violin plots. (A) Long (>100 kb) genes are underrepresented in cold spots relative to control regions and hotspots. (B) Gene expression levels (in transcripts per million reads (TPM)) are similar among regions. (C) Relative to control, open chromatin regions are more common in hotspots and less common in cold spots. **(D, E)** Analysis of CNV location in 15 individuals comprising a previous CNV atlas. (D) 5Mb regions with no CNV overlap (i.e. cold spots) are only observed in real data. (E) Cold spots observed in previous data (outer ring) overlap with LIBD93 cold spots (middle ring).

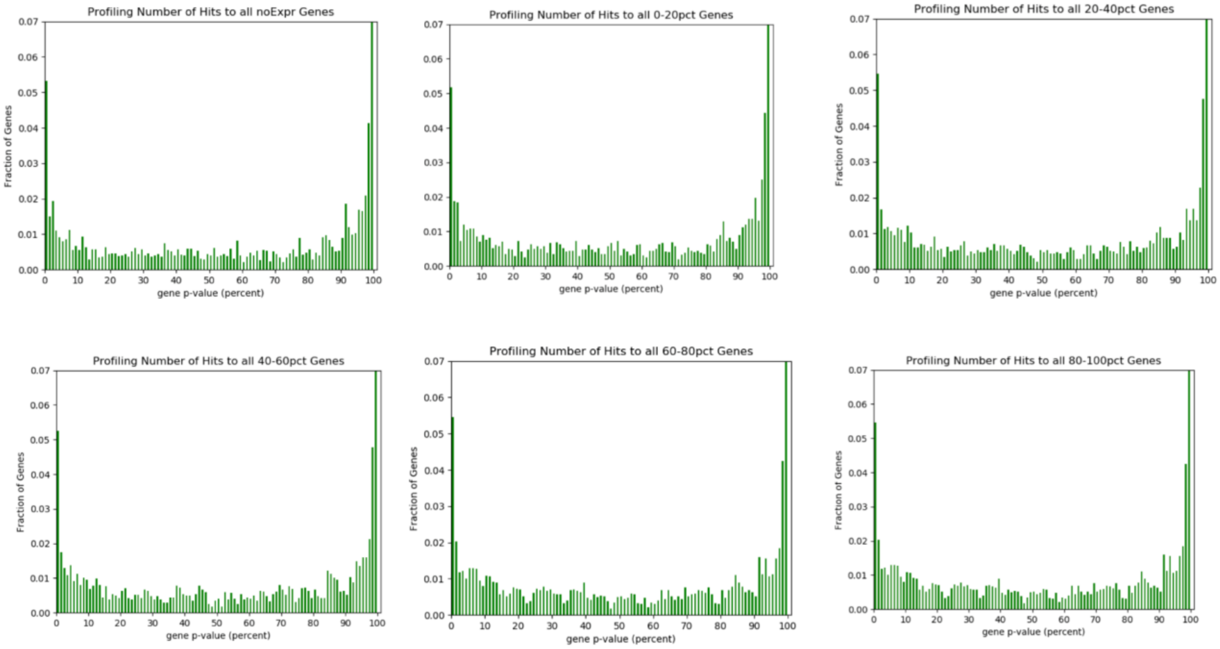

Supplementary Figure 12

Complementary view of hotspots and cold spots in physical genes. Depicted are the *distribution* of p-values (defined as in Supplementary Figure 9 but for physical genes) for genes showing the presence of hotspots (first 5 bins) and cold spots (last bin) in six expression categories (genes not expressed, and 5 quintiles of genes expressed in DLPFC). This analysis is complementary to that performed in 5Mb regions and shows the presence of hotspots and cold spots in genes expressed at various levels.

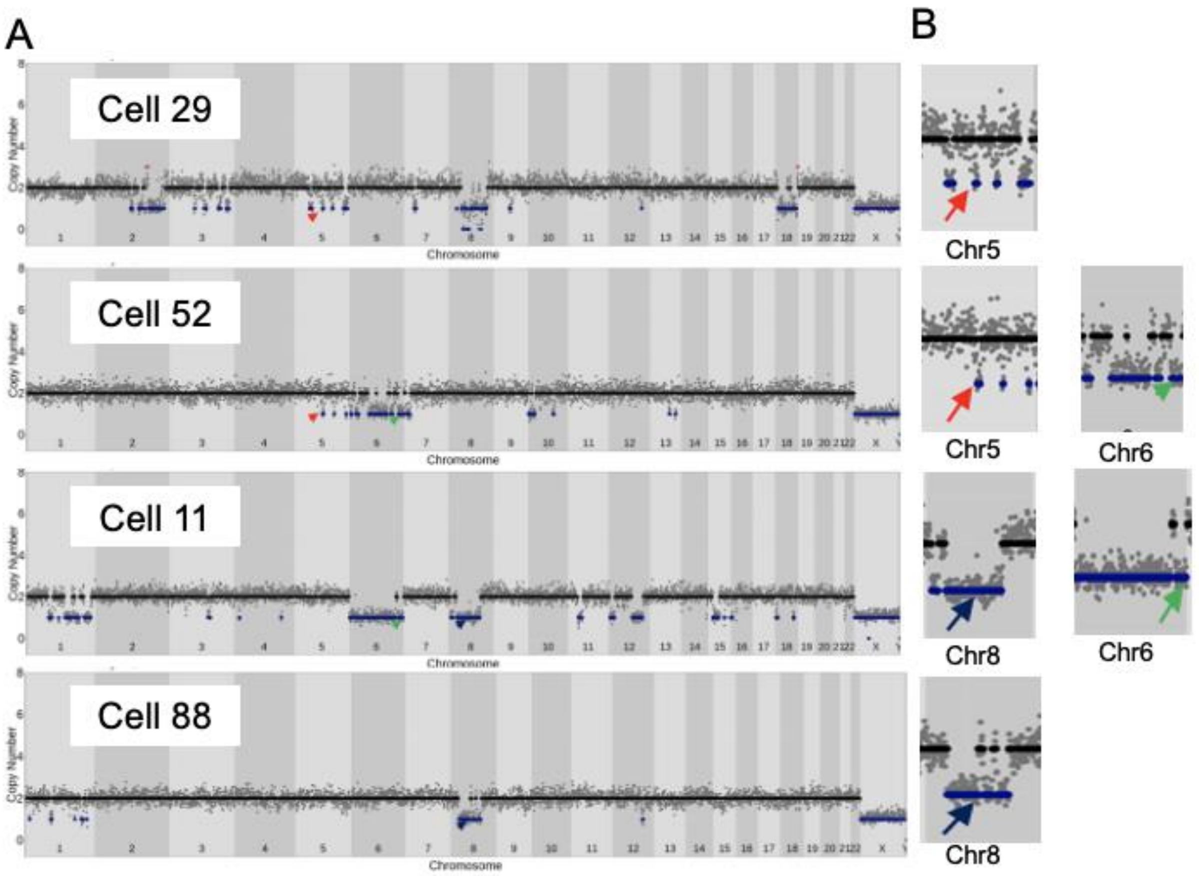

Supplementary Figure 13

Neurons with complex karyotypes and shared CNVBs. (**A,B**) Three pairs of CNVs in 4 cells (shown by red, green and blue arrows respectively) are shared/recurrent. The shared CNVs are magnified in the lower panel. None of the other CNVs are shared. This illustrates that the recurring CNVs are unlikely to be in clonal lineages.
