# Supplementary material for: Mapping recurrent mosaic copy number variation in human neurons
Source: Nat Commun. 2024 May 17;15:4220. doi: 10.1038/s41467-024-48392-0 (PMC11101435; doi:10.1038/s41467-024-48392-0)
Supplement: Supplementary file 3 — Description of Additional Supplementary Files [file 41467_2024_48392_MOESM3_ESM.pdf]

Description of Additional Supplementary Files

File Name: Supplementary Data 1
Description: List of initial predicted somatic CNVs from the application of Ginkgo to 2125 single cell libraries, with *chrom*, *start*, and *end* pertaining to coordinates within the hg19 human reference genome, *CN* indicating the predicting copy number, *barcode* indicating the specific single cell library in which the CNV was detected, *size* indicating its predicated lengths, and *final_call* indicating whether it was included (1) or excluded (2) from the final call set.

File Name: Supplementary Data 2
Description: List of final predicted somatic deletions from the application of Scoval to 2097 filtered single cell libraries, with *chrom*, *start*, and *end* pertaining to coordinates within the hg19 human reference genome, *CN* indicating the predicting copy number, *barcode* indicating the specific single cell library in which the CNV was detected, *size* indicating its predicated lengths, and *cell_id* as a unique simplified label for each barcoded cell.

File Name: Supplementary Data 3
Description: List of predicted somatic CNVs from application of CHISEL to three different sequencing runs. Column labels are primarily defined by the CHISEL output, where *CHR* is the name of a chromosome, *START* is the starting coordinate of a genomic bin, *END* is the ending coordinate of the genomic bin, *CELL* is the barcoded name of a cell, *NORMAL* is the number of sequencing reads from the matched-normal sample for the bin, *COUNT* is the number of sequencing reads from the cell CELL in the bin, *RDR* is the estimated read depth ratio for the bin in the cell CELL, *A-COUNT* is the number of observed sequencing reads from the haplotype A of the SNP, *B-COUNT* is the number of observed sequencing reads from the haplotype B of the SNP, *BAF* is the B-allele frequency estimated for the bin in the cell *CELL*, *CLUSTER* is the cluster where the cell *CELL* has been assigned, *HAP_CN* is the haplotype specific copy number of cell *CELL*, and *CORRECTED_HAP_CN* is the corrected haplotype specific copy number of cell *CELL.* The last column, *overlap*, is an added label to the CHISEL output indicating whether the coordinates overlap with a deletion in the final call set.
