# Supplementary material for: Mapping recurrent mosaic copy number variation in human neurons
Source: Nat Commun. 2024 May 17;15:4220. doi: 10.1038/s41467-024-48392-0 (PMC11101435; doi:10.1038/s41467-024-48392-0)
Supplement: Supplementary file 7 — Reporting Summary [file 41467_2024_48392_MOESM7_ESM.pdf]

nature portfolio

Corresponding author(s): Ryan E. Mills, Michael J. McConnell

Last updated by author(s): March 20, 2024

Reporting Summary

Nature Portfolio wishes to improve the reproducibility of the work that we publish. This form provides structure for consistency and transparency in reporting. For further information on Nature Portfolio policies, see our Editorial Policies and the Editorial Policy Checklist.

Statistics

For all statistical analyses, confirm that the following items are present in the figure legend, table legend, main text, or Methods section.

n/a	Confirmed	
☐	☒	The exact sample size (*n*) for each experimental group/condition, given as a discrete number and unit of measurement
☐	☒	A statement on whether measurements were taken from distinct samples or whether the same sample was measured repeatedly
☐	☒	The statistical test(s) used AND whether they are one- or two-sided<br>*Only common tests should be described solely by name; describe more complex techniques in the Methods section.*
☒	☐	A description of all covariates tested
☒	☐	A description of any assumptions or corrections, such as tests of normality and adjustment for multiple comparisons
☐	☒	A full description of the statistical parameters including central tendency (e.g. means) or other basic estimates (e.g. regression coefficient) AND variation (e.g. standard deviation) or associated estimates of uncertainty (e.g. confidence intervals)
☐	☒	For null hypothesis testing, the test statistic (e.g. *F*, *t*, *r*) with confidence intervals, effect sizes, degrees of freedom and *P* value noted<br>*Give P values as exact values whenever suitable.*
☐	☒	For Bayesian analysis, information on the choice of priors and Markov chain Monte Carlo settings
☒	☐	For hierarchical and complex designs, identification of the appropriate level for tests and full reporting of outcomes
☒	☐	Estimates of effect sizes (e.g. Cohen's *d*, Pearson's *r*), indicating how they were calculated

Our web collection on statistics for biologists contains articles on many of the points above.

Software and code

Policy information about availability of computer code

Data collection	no software used
Data analysis	The workflow to generate the final call set is available at https://github.com/mills-lab/Scoval.

For manuscripts utilizing custom algorithms or software that are central to the research but not yet described in published literature, software must be made available to editors and reviewers. We strongly encourage code deposition in a community repository (e.g. GitHub). See the Nature Portfolio guidelines for submitting code & software for further information.

Data

Policy information about availability of data

All manuscripts must include a data availability statement. This statement should provide the following information, where applicable:

- Accession codes, unique identifiers, or web links for publicly available datasets
- A description of any restrictions on data availability
- For clinical datasets or third party data, please ensure that the statement adheres to our policy

Data and call sets have been deposited in the NIMH Data Archive (NDA Study ID 1680, http://dx.doi.org/10.15154/1527774) and can be accessed as part of the NIMH Data Archive permission groups: https://nda.nih.gov/user/dashboard/data_permissions.html.

Research involving human participants, their data, or biological material

Policy information about studies with human participants or human data. See also policy information about sex, gender (identity/presentation), and sexual orientation and race, ethnicity and racism.

Reporting on sex and gender	1 Male, no sex or gender studies were performed
Reporting on race, ethnicity, or other socially relevant groupings	1 African Ancestry, no grouping or comparison analyses were performed
Population characteristics	No population was analyzed
Recruitment	Donor was identified through the Medical examiner in Baltimore County. Consent for donation was given by next-of-kin.
Ethics oversight	Postmortem human brain tissue was obtained at the time of autopsy via audiotaped witnessed informed consent from the legal next-of-kin, through the Office of the Chief Medical Examiner of the State of Maryland, under the following two protocols: Maryland Department of Health IRB protocol #12–24 and the WCG protocol #20111080.

Note that full information on the approval of the study protocol must also be provided in the manuscript.

Field-specific reporting

Please select the one below that is the best fit for your research. If you are not sure, read the appropriate sections before making your selection.

☒ Life sciences ☐ Behavioural & social sciences ☐ Ecological, evolutionary & environmental sciences

For a reference copy of the document with all sections, see nature.com/documents/nr-reporting-summary-flat.pdf

Life sciences study design

All studies must disclose on these points even when the disclosure is negative.

Sample size	2025 neurons from one individual. No power calculations were performed. Past studies analyzed at most ~120 neurons per individual, in this individual we sought to exceed this by > 10-fold.
Data exclusions	Our quality control (QC) filters excluded 28 single neurons with aberrant bin-to-bin variance [i.e., Median Absolute Deviation (MAD), 2097 (>95%) libraries passed QC] and masked 308 genomic bins that were outliers in global read coverage across all neurons (Supplementary Fig. 1B-D).
Replication	Single cells are destroyed when their DNA is measured. Thus, repeated measures of one cell's genome is not possible. We attempted to replicate our findings of hotspots and cold spots in a previously published data set. The neuronal CNV atlas assembled in Chronister, et al. (2019) Cell Reports. This atlas contains only 867 neurons and represents a composite of 15 individuals ranging from <1 year-old to >90 years-old. Although hotspots may vary by individual and CNV neuron frequency declines with age, we found that cold spots in the CNV atlas also cluster on few chromosomes and 40% of these overlap cold spots identified in this study (Supplementary Fig. 11E).
Randomization	No experimental groups were studied.
Blinding	Sample blinding was not relevant because one individual was studied.

Reporting for specific materials, systems and methods

We require information from authors about some types of materials, experimental systems and methods used in many studies. Here, indicate whether each material, system or method listed is relevant to your study. If you are not sure if a list item applies to your research, read the appropriate section before selecting a response.

Materials & experimental systems

n/a	Involved in the study
☐	☒ Antibodies
☒	☐ Eukaryotic cell lines
☒	☐ Palaeontology and archaeology
☒	☐ Animals and other organisms
☒	☐ Clinical data
☒	☐ Dual use research of concern
☒	☐ Plants

Methods

n/a	Involved in the study
☒	☐ ChIP-seq
☐	☒ Flow cytometry
☒	☐ MRI-based neuroimaging

Antibodies

Antibodies used	mouse monoclonal anti-human NeuN IgG Alexa Fluor 555 Conjugate clone A60<br>EMD via Millipore Cat. # MAB377A5. No secondary antibody was required.
Validation	https://www.emdmillipore.com/US/en/product/Anti-NeuN-Antibody-clone-A60-Alexa-Fluor-555-Conjugate,MM_NF-MAB377A5#anchor_REF<br>Both FACS and fluorescence microscopy identified clear positive and negative populations.

Plants

Seed stocks	*Report on the source of all seed stocks or other plant material used. If applicable, state the seed stock centre and catalogue number. If plant specimens were collected from the field, describe the collection location, date and sampling procedures.*
Novel plant genotypes	*Describe the methods by which all novel plant genotypes were produced. This includes those generated by transgenic approaches, gene editing, chemical/radiation-based mutagenesis and hybridization. For transgenic lines, describe the transformation method, the number of independent lines analyzed and the generation upon which experiments were performed. For gene-edited lines, describe the editor used, the endogenous sequence targeted for editing, the targeting guide RNA sequence (if applicable) and how the editor was applied.*
Authentication	*Describe any authentication procedures for each seed stock used or novel genotype generated. Describe any experiments used to assess the effect of a mutation and, where applicable, how potential secondary effects (e.g. second site T-DNA insertions, mosiacism, off-target gene editing) were examined.*

Flow Cytometry

Plots

Confirm that:

☒ The axis labels state the marker and fluorochrome used (e.g. CD4-FITC).

☒ The axis scales are clearly visible. Include numbers along axes only for bottom left plot of group (a 'group' is an analysis of identical markers).

☒ All plots are contour plots with outliers or pseudocolor plots.

☒ A numerical value for number of cells or percentage (with statistics) is provided.

Methodology

Sample preparation	We labeled nuclei derived from neurons by incubating with mouse monoclonal anti-human NeuN IgG clone A60 (Alexa Fluor 555 conjugate) diluted 1:250 in blocking buffer overnight at 4°C. We verified that NeuN+ nuclei also contained dsDNA by co-staining with either SYTO 13 green fluorescent nucleic acid stain at 500 nM
Instrument	Becton Dickenson Influx Cell Sorter
Software	BD FACSdiva 8.0.1
Cell population abundance	110,000 events were detected. 12,316 of these were singlets. 33.9% of these were NeuN+.
Gating strategy	Nuclei were identified based on DNA staining. These were gated to singlets based on forward and side scatter width.

☒ Tick this box to confirm that a figure exemplifying the gating strategy is provided in the Supplementary Information.
